# Moving Particles Through a Finite Element Mesh

**DOI:** 10.6028/jres.103.004

**Published:** 1998-02-01

**Authors:** Adele P. Peskin, Gary R. Hardin

**Affiliations:** National Institute of Standards and Technology, Boulder, CO 80303

**Keywords:** finite element, moving boundary, packed beds, particles

## Abstract

We present a new numerical technique for modeling the flow around multiple objects moving in a fluid. The method tracks the dynamic interaction between each particle and the fluid. The movements of the fluid and the object are directly coupled. A background mesh is designed to fit the geometry of the overall domain. The mesh is designed independently of the presence of the particles except in terms of how fine it must be to track particles of a given size. Each particle is represented by a geometric figure that describes its boundary. This figure overlies the mesh. Nodes are added to the mesh where the particle boundaries intersect the background mesh, increasing the number of nodes contained in each element whose boundary is intersected. These additional nodes are then used to describe and track the particle in the numerical scheme. Appropriate element shape functions are defined to approximate the solution on the elements with extra nodes. The particles are moved through the mesh by moving only the overlying nodes defining the particles. The regular finite element grid remains unchanged. In this method, the mesh does not distort as the particles move. Instead, only the placement of particle-defining nodes changes as the particles move. Element shape functions are updated as the nodes move through the elements. This method is especially suited for models of moderate numbers of moderate-size particles, where the details of the fluid-particle coupling are important. Both the complications of creating finite element meshes around appreciable numbers of particles, and extensive remeshing upon movement of the particles are simplified in this method.

## 1. Introduction

### 1.1 Background

Fluid-solid systems are classified based on the size and number of particles (or other objects). Particle sizes range from aerosols to large objects. The focus of an analysis may be the detailed movement of a single particle, or the interaction of a large number of objects. Different numerical techniques are appropriate for different classes of problems. In systems with very small particles, such as aerosols, the number of particles is typically very large. In these systems the effect of the particles on the flow is often ignored. Corrections can be made to account for the momentum transferred to the particles, but there are too many particles to consider the details of the coupled dynamical interaction between the fluid and the particles.

Details of the fluid-particle interactions are important in systems such as fluidized beds, steam turbines, natural gas pipelines, and sprayers. In such systems it is important to know how particles interact to affect their trajectories, which is seen most clearly when particles make contact with one another. A simulation must couple the dynamics of the fluid and the particles to achieve an accurate solution.

One approach for such systems is to mesh the fluid surrounding the particles, taking into account the interior boundaries created by the particles. Such meshes are necessarily complex because of the large curvature of the particle boundary compared with the boundaries of the larger domain. Consequently, it is convenient to use unstructured meshes. The ability to use unstructured grids is a potential advantage of finite element methods compared to finite difference techniques. When the particle or particles move, the mesh is allowed to distort to some degree. This distortion tends to reduce the accuracy of the simulation. After the particle(s) travel some distance the distortion becomes too great, and either the entire domain or a region around each particle is remeshed.

Tezduyar and collaborators [1–5] are developing efficient algorithms for moving the particles and updating the mesh. They have developed what they call a Deforming-Spatial-Domain/Space-Time procedure for simulating moving particles in two or three dimensions. Triangular (or tetrahedral in 3D) elements are used to discretize the fluid phase surrounding the particles. Special narrow elements are used near each particle to track the boundary layer. The elements near each particle deform as the particle moves through the mesh. Remeshing is necessary when the elements become overly distorted. Both remeshing and checking the mesh for distortion can be computationally intensive processes if they are done at each time step. Tezduyar et al. discuss that this is particularly true for computations on parallel-architecture machines because of the time required to apportion the meshing tasks among the processors. Consequently, remeshing is typically performed only at specified timesteps. This creates a potential for increased error if too much distortion occurs between remeshing. As a result, it appears that this technique is most suitable for slowly deforming spatial domains. One means they employ to limit the extent to which remeshing is necessary is to translate the entire mesh at the velocity of the center of mass of the set of spheres.

Hu, Joseph, and Crochet [6] describe a method for direct simulation of flowing “particles” in two dimensions, using unstructured triangular finite element meshes. At each time step they remesh, generating a new mesh that bears no straightforward relationship to the previous mesh. This then requires the regeneration of element-connectivity information, which is necessary for the finite element method to know how the solution interacts between adjacent elements. This is facilitated by a new mesh-data structure that can be processed more efficiently to find the position of a given node. The solution from the previous time step must be interpolated onto the new mesh. Their method can track computational domains that move drastically with time. They use a stable time-integration scheme in which “at each time step, the positions of the particles are updated explicitly, the computational domain is remeshed, the solution at the previous time is mapped onto the new mesh, and finally the nonlinear Navier-Stokes equation and the implicitly discretized Newton’s equations for particle velocities are solved on the new mesh iteratively” [6]. The difficulties they address are remeshing at each time step, the interpolation of the solution onto the new mesh, and finally the general issue of solving the system in a stable and efficient manner.

A significant issue of both of these techniques is the establishment of robust criteria for acceptable vs unacceptable distortion between remeshing. Remeshing can be computationally intensive, particularly in three dimensions, and especially for parallel computations. Error is introduced every time the solution at a given time step must be projected onto a new mesh. While approaches involving unstructured meshes with remeshing have proved to work well for at least certain sets of problems, we were motivated to seek a technique that would avoid the computational difficulties of remeshing, and interpolation of the solution onto new meshes.

Our method was motivated by that of Fogelson and Peskin [7]. They developed a fast finite difference method for solving Stokes’ equations in three dimensions to describe the motions of elastically deformable suspended particles (rigid particles are modeled as slightly deformable). The domain is discretized on a regular lattice. They represent each particle by a separate set of points overlying the lattice. Equations of fluid motion are applied at all points on the lattice, including those overlain by particles. The particle nodes move at the local fluid velocity. However, the local fluid velocity is affected by the presence of the particle through a force-density term that accounts for the resistance of the particles to deformation. It affects the fluid near the particle. The particles are modeled as regions of the fluid with elastic cohesive forces. This allows them to solve the entire domain as a single phase, with no fluid-solid boundaries introduced by the particles. The result is a very fast technique at the expense of some accuracy about the fluid-particle interaction. In addition to the internal elastic links, any external forces are also handled in a straightforward way, including interparticle forces. Since this is a purely Stokes model, it does not account for the inertia of the particle or the fluid.

Unverdi and Tryggvason [8] briefly survey different methods of modeling sharp fronts that illustrates the breadth of different techniques that are available. In this paper they use an approach that is somewhat similar to that of Fogelson and Peskin. However, it tracks the interface explicitly, which allows them to consider different phases as distinct. To look at the motion of bubbles in a fluid, they use a regular finite difference grid onto which they overlay an unstructured grid that represents the particle surface. The interface region between the particle surface and the surrounding fluid is given a thickness, on the order of the mesh spacing, to allow a more gradual transition between the phases. This is done to provide stability and smoothness. The main advantage of this method is its ability to track multiple interfacial boundaries in the same region of the grid.

### 1.2 A New Technique

Our work follows a somewhat different approach. We wanted to avoid the difficulty of remeshing a significant fraction of the domain. This led us to an approach using overlying sets of nodes to represent the particles. We also wanted to be able to render the solution around a particle as accurately as possible. In adapting this approach to finite elements it appeared natural to make the extra nodes belong to the elements in which they appear. We have developed a dynamic finite element type in which the shape functions used on a given element are allowed to change in time as a particle boundary moves into and out of an element.

A background mesh is designed to fit the geometry of the overall domain. The mesh is designed independently of the presence of the particles except in terms of how fine the mesh must be to track particles of a given size. The particles are represented by circles which overlie the mesh. Nodes are added to the mesh where the particle boundaries intersect the (background) mesh, increasing the number of nodes contained in each element whose boundary is intersected. These additional nodes are then used to describe and track the particle in the numerical scheme. Appropriate element shape functions are defined to approximate the solution on the elements with extra nodes. The particles are moved through the mesh by moving only the extra, overlying nodes defining the particles. Otherwise, the mesh remains unchanged: the background mesh does not distort as the particles move. Element shape functions are dependent on the placement of the particle nodes with respect to the element sides, and change dynamically as the particles move through the mesh. Shape functions are updated as the particle nodes move through the elements. We demonstrate our technique with flow examples for which exact solutions are available.

A primary advantage of this method is that there is no mesh distortion as particles move. This is significant because remeshing tends to be time-consuming and error-prone. We also avoid extensive checking for whether remeshing is necessary. The particles intersect underlying elements at different locations as they move, and the element shape functions change to account for this movement. The only additional storage required is the current location of the particle nodes, and their relative position along an element side. At each time step of a transient analysis, the translation and rotation of each particle are found directly from the continuum equations for the fluid, and Newton’s equations for the particles, and the nodes that represent that particle are moved accordingly. Specifically, intersection points where particle boundaries meet element edges, and variables describing positions along element edges are updated. The method is easily extended to particles with irregular shapes, and different shapes for different particles.

The various methods described above are best suited to different classes of problems. The first approach, in which the mesh is allowed to distort, is well suited to the accurate solution of a small number of particles. With larger numbers of particles there is a temptation to remesh less often because of the expense, which compromises accuracy. The finite difference approach using an overlying mesh is well suited for large numbers of particles, especially when the details of the flow near the particles are not as important. Our method avoids the complications of creating finite element meshes around appreciable numbers of particles, and the extensive remeshing required to track the movement of particles is eliminated. At the same time, it incorporates the particle boundaries directly into the mesh, allowing for an accurate solution near the particles. This tends to make this technique more appealing for a system with a moderate number of particles, which cover most of the domain.

If large areas of the domain contain no particles, then computational resources are unnecessarily concentrated there. Nonetheless, we believe that this technique, or an extension of it, may also prove to be useful for systems involving smaller numbers of particles. A possible extension is simply to add extra “rings” or layers of nodes around the moving objects as needed to accurately represent the solution, so that the node density is increased primarily only in localized regions around the particles. This would also be very useful for modeling flows at higher Reynolds numbers, where the local fluid-particle interactions are more complex. Another possibility is to refine the mesh locally around each particle in a prescribed way. These well-defined “refinement zones” would move with the particles, but the background mesh would remain undistorted.

## 2. Equations of Motion

### 2.1 Flow Equations

The equations describing conservation of momentum and mass are as follows. (For definitions of the various quantities, see the glossary.)

Momentum equation:

$$\rho_{0}\left(\frac{\partial u}{\partial t}+u\cdot \nabla u\right)=-\nabla p+\mu \nabla^{2}u+\rho g,$$ (1)

where ***u*** = (*u_x_*, *u_y_*, *u_z_*).

Continuity equation:

∇⋅u=0. (2)

A standard finite element methodology for incompressible flow is to replace the continuity equation with a “penalty” constraint (as described in Hughes et al. [9]), in order to eliminate the need to solve for the “pressure” field directly. This is often useful, as long as the particle boundary conditions are not direct functions of pressure.

Penalty equation:

∇⋅u=−ϵp. (3)

Our code also has other capabilities, such as modeling of heat transfer and chemical reactions, that are of interest in a fluid-particle simulation. These capabilities are easily extended to the particles and particle boundaries. However, they are not germane to the present discussion, so for clarity we omit them. Boundary conditions for the particles prescribe no slip at the fluid-particle interface.

Boundary conditions:

$$u_{\text{fluid}}=u_{\text{solid}}\;\text{at praticle boundaries}.$$ (4)

Presently, we are considering only solid particles, but this technique should be readily extensible to fluid drops or bubbles, yielding a two-phase fluid-fluid system.

### 2.2 Equations to Represent Particle Movement

For the purposes of developing and testing this technique we are presently working only on two-dimensional flow. This is a constraint of practicality for development. It is straightforward to extend the method to three-dimensional flows. The movement of a 2-D “particle” is determined by three degrees of freedom: two translational and one rotational. All nodes defining an individual particle are constrained to have the same translational and rotational movement. Components for rotation vary according to their position on the circle representing the boundary of the particle: each has the same speed, but a different direction of movement in a Cartesian sense. The net velocity of each particle node is a sum of these contributions. An additional constraint, conservation of angular momentum, is added to the set of global equations for each particle corresponding to the rotational degree of freedom. Particles conserve both linear and angular momentum.

Conservation of linear momentum:

$$m_{p}g+\int \left(n\cdot \sigma \right)ds=m_{p}\left(\frac{\partial u_{x},u_{y}}{\partial t}\right).$$ (5)

Conservation of angular momentum:

$$\int \left(r\times \left(n\cdot \sigma \right)\right)=\left(\frac{\partial w}{\partial t}\right)I.$$ (6)

**Fig. 1:**
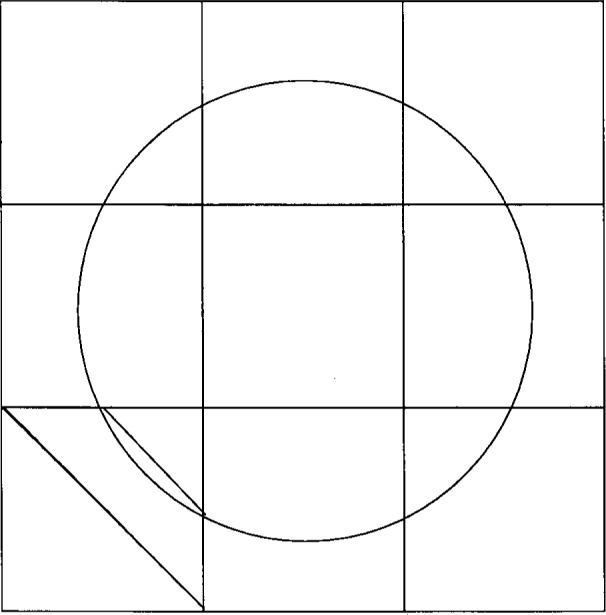
Construction of a sub-element for the computation of a normal to a particle.

To evaluate these constraints on each particle, we define one-dimensional line elements along the particle boundary. Each line element lies within a two-dimensional four-node element of the background mesh. Within this element is constructed a separate two-dimensional four-node “sub-element” associated with the particular line element. This new sub-element is handled exactly like any other four-node element, but is formed solely for the purpose of computing normal gradients of the velocity along the line element, for computation of the stress boundary condition. Two of the corner nodes of this sub-element are the end-points of the line element, which are the intersections of the particle boundary with the particular background element. The other two corner nodes are the adjacent corner nodes of the background element. Thus, two of the sides of the sub-element lie along sides of the background element, one side approximates the boundary of the particle that overlaps the background element, and the fourth side of the sub-element is a diagonal of the background element. This construction is illustrated in Fig. 1.

## 3. Variable Finite Element Shape Functions

As discussed above, we are currently limited to two-dimensional simulations. We are using bilinear (straight-sided) 4-node quadrilateral elements for the background mesh. We superpose on this “background” mesh circles that represent the outlines of particles. This is generalizable to somewhat irregular shapes. Representation of highly contorted shapes will require further development. We add an extra node at each intersection of a circle with an element side. For a given 4-node element, we consider cases where the particle intersects the elements edges at either 2 or 4 points. It is very rare for the particle to intersect an element at exactly 1 or 3 points, because the time dimension is finite differenced, and consequently, discrete. Intersection at 1 or 3 points would require the boundary of the particle to be exactly tangent to an element side. Numerically the particles move in small increments, so the chances of the edges coinciding tangentially to within machine accuracy are minute. Currently, we simply ignore such points, assuming they do not coincide. As yet, we have not observed exact coincidence to occur.

We developed shape functions for the resultant 6-node or 8-node elements, based on the method described in Zienkiewicz [10] for generating “serendipity” elements. In the finite element method, a solution to the transport equations for a given dependent variable is represented over an element by summing the contribution from each shape function, corresponding to each node, over all of the nodes of the element. Lagrangian interpolation functions are generally used as the shape functions because they yield the convenient property that the nodal values of the dependent variables are available directly, without summing contributions. For example, temperature on an element, *T*, would be represented by:

$$T=\sum_{i}\phi_{i}T_{i}.$$ (7)

*T_i_* factors are coefficients (constant at a given timestep) corresponding to the temperature at each node *i* of the element, and the *ϕ_i_* are the nodal basis functions.

**Fig. 2:**
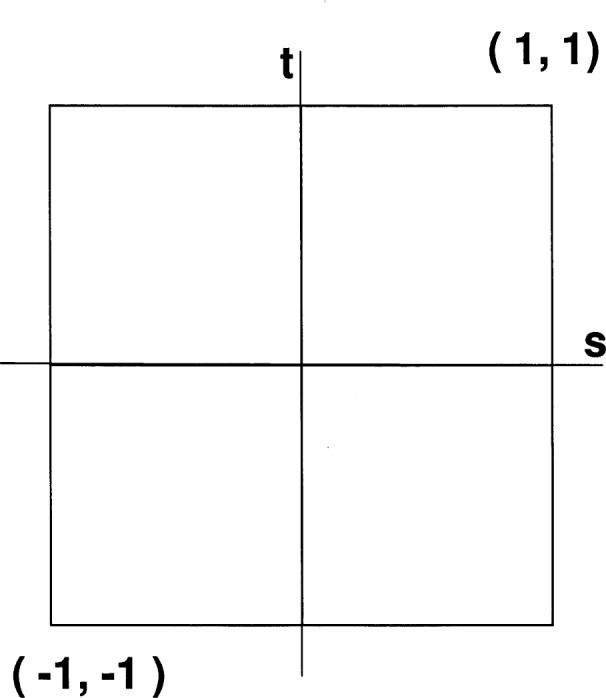
The parent element, onto which all elements in the physical domain are mapped.

Following standard finite element methodology, each element on the “physical” domain (the mathematical representation of the physical domain), on which the problem is posed, is mapped locally onto a regular 2 × 2 square element, called the “parent” element, centered around the point (0,0) (see Fig. 2). This allows the use of one set of standard shape functions, which is defined on this unchanging “parent”. The representation of the solution on each element is actually formulated on this parent element. We designate a Cartesian coordinate system on this parent element as {*s*, *t*}. Both *s* and *t* range from −1 to 1. In this system, the shape functions for a bilinear 4-node element, where the nodes are numbered counterclockwise starting at the lower left, are defined below.

Standard bilinear finite element shape functions for a quadrilateral element are:

$$N_{1}=\frac{1}{4}\left(1-s\right)\left(1-t\right),$$ (8)

$$N_{2}=\frac{1}{4}\left(1+s\right)\left(1-t\right),$$ (9)

$$N_{3}=\frac{1}{4}\left(1+s\right)\left(1+t\right),$$ (10)

$$N_{4}=\frac{1}{4}\left(1-s\right)\left(1+t\right),$$ (11)

Each function has a value of 1 at the node for which it is defined, and a value of 0 at all other node points. This is the property that allows the solution at the nodes to be obtained directly. When new nodes are added, care must be taken to adjust the shape functions at all affected nodes to account for this. Otherwise, unintended contributions are erroneously added to the representation, distorting the solution. Again, this is standard finite element methodology. The novelty lies in the use of the new elements to track the particle boundaries.

For elements that contain extra nodes, for each additional node an additional bilinear shape function is added to the representation of the solution on that element. The specific shape function that is added for each extra node is based on the position of that node along the side, and depends on which side of the element it is on. Shape functions for the end nodes (corner nodes) of sides containing an extra node must be modified. Additional terms are added to them to ensure that their shape function value is zero at the new nodes as well as the other “original” (here, corner) nodes in the element. This is an important facet of the finite element approach based on Lagrangian polynomials. The requirement is that at each node of an element only one shape function contributes, the one corresponding to that node, and the value of the shape function is 1. With this formulation, the value of a dependent variable at a node is simply the value of the coefficient of the shape function for that node. This avoids any need to compute an inverse mapping between the “parent” element on the locally mapped domain and the “physical” element on the unmapped domain. It also eliminates the need to sum the polynomials representing the solution on each element. The computation of the nodal solutions is direct (see Zienkiewics [10].)

For the new, 6-node element, two new shape functions are needed corresponding to the two new nodes. Consider, for example, the case with a particle node along the bottom (side 1) and another on the right side (side 2). These new nodes are designated nodes 5 and 6, respectively. Let *p* represent the *s* value of the extra node along side 1, and *q* represent the *t* value of the extra node along side 2. The new nodes are located at (*p*, −1) and (1, *q*). The shape functions for each of the extra nodes is piecewise continuous.

New bilinear finite element shape functions for particle nodes are:

$$N_{5}=\{\begin{array}{cc} \frac{\left(1-t\right)\left(1+s\right)}{2\left(1+p\right)} & \text{for}\;s\leq p\\ \frac{\left(1-t\right)\left(1-s\right)}{2\left(1-p\right)} & \text{for}\;s>p, \end{array}$$ (12)

$$N_{6}=\{\begin{array}{cc} \frac{\left(1+s\right)\left(1+t\right)}{2\left(1+q\right)} & \text{for}\;t\leq q\\ \frac{\left(1+s\right)\left(1-t\right)}{2\left(1-q\right)} & \text{for}\;t>q. \end{array}$$ (13)

At points 5 and 6 the values of shape functions *N*_1_ – *N*_4_ must equal 0. Consequently, these four shape functions must be changed according to the location of the extra node.

The modified bilinear finite element shape functions for corner nodes of a particle element are:

$$N_{1}^{\prime }=N_{1}-\frac{1}{2}N_{5}\left(1-p\right),$$ (14)

$$N_{2}^{\prime }=N_{2}-\frac{1}{2}N_{5}\left(1+p\right)-\frac{1}{2}N_{6}\left(1-q\right),$$ (15)

$$N_{3}^{\prime }=N_{3}-\frac{1}{2}N_{6}\left(1+q\right),$$ (16)

$$N_{4}^{\prime }=N_{4}\;\left(\text{unchanged}\right).$$ (17)

The entire set of six shape functions on the new parent element is:

$$N_{1}=\frac{1}{4}\left(1-s\right)\left(1-t\right)-\frac{1}{2}N_{5}\left(1-p\right),$$ (18)

$$N_{2}=\frac{1}{4}\left(1+s\right)\left(1-t\right)-\frac{1}{2}N_{5}\left(1+p\right)-\frac{1}{2}N_{6}\left(1-q\right),$$ (19)

$$N_{3}=\frac{1}{4}\left(1+s\right)\left(1+t\right)-\frac{1}{2}N_{6}\left(1+q\right),$$ (20)

$$N_{4}=\frac{1}{2}\left(1-s\right)\left(1+t\right),$$ (21)

$$N_{5}=\{\begin{array}{cc} \frac{\left(1-t\right)\left(1+s\right)}{2\left(1+p\right)} & \text{for}\;s\leq p\\ \frac{\left(1-t\right)\left(1-s\right)}{2\left(1-p\right)} & \text{for}\;s>p, \end{array}$$ (22)

$$N_{6}=\{\begin{array}{cc} \frac{\left(1+s\right)\left(1+t\right)}{2\left(1+q\right)} & \text{for}\;t\leq q\\ \frac{\left(1+s\right)\left(1-t\right)}{2\left(1-q\right)} & \text{for}\;t>q. \end{array}$$ (23)

We use a subparametric mapping from each “particle element” (each element containing part of a particle) in the physical domain to the parent element: more nodes are used to approximate the solution on a particle element than are used to define the mapping. This is done to preserve a linear mapping, to avoid unnecessary distortion in moving from the physical domain to the locally mapped domain. This is sensible, since in this technique the movement of the particles does not cause any distortion of the elements of the background mesh. Only the four corner nodes are necessary to define the Jacobian of the transformation from the coordinate system on the element in physical domain to (*s*, *t*) coordinates on the parent. The values of *p* and *q* are updated following each step in a transient analysis with moving particles. There are significantly fewer steps involved in updating the mesh than in remeshing schemes. (See, for example, Johnson et al. [1] and Hu et al. [6] for discussions of particular mesh-updating strategies.)

For cases in which an edge of a particle moves very close to an element side, there can be two intersections of the particle and the element along the same edge. In that case a slightly different set of linear shape functions is defined for that element. Suppose there is a resulting 6-node element with 2 extra nodes along side 1 whose *s* coordinates have values of *p* and *q*. Assuming *p* is less than *q*, let *p*_1_ = (*p* − 1)/2, *q*_1_ = (*q* + 1)/2. The corresponding six shape functions are:

$$N_{1}=\frac{1}{4}\left(1-s\right)\left(1-t\right)-\frac{1}{2}N_{5}\left(1-p\right)-\frac{1}{2}N_{6}\left(1-q\right),$$ (24)

$$N_{2}=\frac{1}{4}\left(1+s\right)\left(1-t\right)-\frac{1}{2}N_{5}\left(1+p\right)-\frac{1}{2}N_{6}\left(1+q\right),$$ (25)

$$N_{3}=\frac{1}{4}\left(1+s\right)\left(1+t\right),$$ (26)

$$N_{4}=\frac{1}{4}\left(1-s\right)\left(1+t\right),$$ (27)

$$N_{5}=\{\begin{array}{cc} -\frac{\left(s+1\right)\left(t-1\right)}{2\left(p+1\right)} & \text{for}\;s<p\\ -\frac{\left(s-q\right)\left(t-1\right)}{2\left(p-q\right)} & \text{for}\;p<s<q\\ -\frac{\left(s-q\right)\left(t-1\right)}{4\left(q-1\right)} & \text{for}\;q<s<q_{1}\\ -\frac{\left(1-s\right)\left(t-1\right)}{4\left(q-1\right)} & \text{for}\;q_{1}<s, \end{array}$$ (28)

$$N_{6}=\{\begin{array}{cc} \frac{\left(s+1\right)\left(t-1\right)}{4\left(p+1\right)} & \text{for}\;s<p_{1}\\ -\frac{\left(s-p\right)\left(t-1\right)}{4\left(p+1\right)} & \text{for}\;p_{1}<s<p\\ \frac{\left(s-p\right)\left(t-1\right)}{2\left(p-q\right)} & \text{for}\;p<s<q\\ -\frac{\left(s-1\right)\left(t-1\right)}{2\left(q-1\right)} & \text{for}\;q<s. \end{array}$$ (29)

(*N*_5_ and *N*_6_ correspond to the two extra nodes on side 1.)

We have incorporated this moving-boundary method into our own software, which we wrote in C++ for simulating and analyzing fluid flow with associated heat and mass transfer. It was written with an object-oriented design in order to facilitate adding new features, such as new moving-boundary methods, new sets of global equations, new boundary conditions, etc. For additional information about our software, see Peskin and Hardin [11].

All of the simulations presented in this paper are two-dimensional representations of flows. We are currently working on a three-dimensional version of this method, which is a straightforward extension of the two-dimensional case. Eight-node brick elements are used for the finite element mesh. A single element can be totally enveloped in a spherical particle, or partially enveloped. When only a portion of a brick element contains part of a sphere, the sphere intersects that element along either 3 or 4 of the brick edges in the most common cases. Analogous to the 2-D cases, there are other, less-common possibilities that should also be accounted for. Different sets of shape functions are defined for elements corresponding to different types of sphere-particle overlap.

## 4. Test Problems

We present simulations to test this moving-boundary technique. We first compare our numerical solutions to exact solutions for a set of Stokes-flow problems. We then present a solution of Navier-Stokes flow around multiple particles in a channel.

Flow patterns around circular two-dimensional “particles” represent cross-sections of flows around cylinders. We present solutions for Stokes flow past a stationary cylinder (or, from another perspective, flow around a translating cylinder), the flow generated by a cylinder rotating at a constant angular velocity, and flow past a rotating cylinder (or, flow around a cylinder that is both rotating and translating). For each of these problems we have looked at the improvement in accuracy of the solution as the finite element mesh is refined.

### 4.1 Stokes Flow Past a Stationary Cylinder

An exact solution exists for Stokes flow around a cylinder. The cylinder is surrounded by a fluid of infinite extent. Far upstream of the cylinder the flow approaches a constant one-dimensional velocity. The stream function for this flow is given by [12]:

$$\psi =v_{0}y\left(1-\frac{R^{2}}{{\left(x^{2}+y^{2}\right)}^{1/2}}\right).$$ (30)

Velocity components follow from the *x* and *y* derivatives of the stream function:

$$u_{x}=v_{0}\left(1-\frac{R^{2}}{{\left(x^{2}+y^{2}\right)}^{1/2}}+\frac{y^{2}R^{2}}{{\left(x^{2}+y^{2}\right)}^{3/2}}\right),$$ (31)

$$u_{y}=v_{0}\frac{xyR^{2}}{{\left(x^{2}+y^{2}\right)}^{3/2}}.$$ (32)

**Fig. 3:**
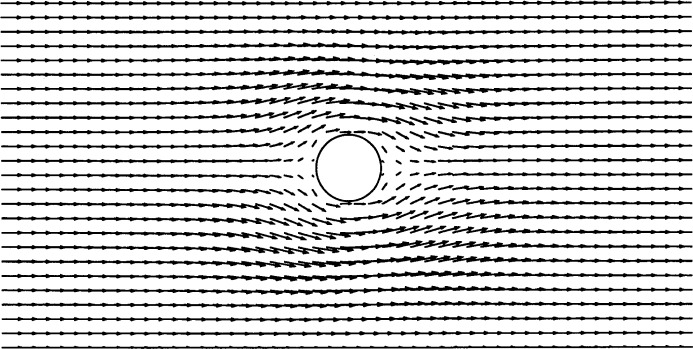
Flow over a stationary cylinder—1250-node mesh.

We created a regular, 1250-node (50 × 25) finite element mesh for this problem, and placed a particle at the center of the mesh. The flow was set equal to a constant value (*U*_∞_) at the boundaries of the mesh. The Reynolds number based on *U*_∞_ was *Re* = 0.1. The resulting flow field is shown in Fig. 3. The same application was run with a 2450-node (70 × 35) mesh and a 3200-node (80 × 40) mesh. The root-mean-square (RMS) errors in the numerical solutions for the three meshes are, respectively, 5.059 %, 4.841 %, and 3.022 %.

**Fig. 4:**
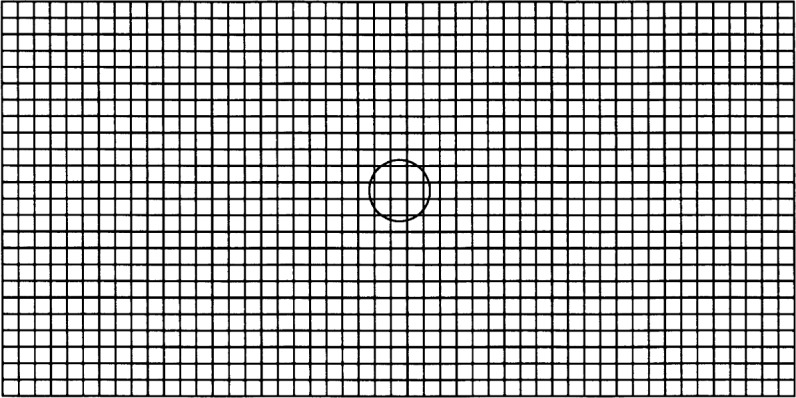
1250-node finite element mesh with a particle.

Separate errors were computed for each velocity component to better gauge the accuracy of the technique: this gives information on the accuracy of the directions as well as magnitudes of the velocity vectors. As expected, the errors decrease as the mesh is refined. Errors for the *x*-component in the three meshes were 5.536 %, 5.127 %, and 3.070 %, and for the *y*-component, 2.514 %, 1.992 %, and 2.268 %. The 1250-node mesh is shown in Fig. 4, with the particle used in this simulation placed at the center.

**Fig. 5:**
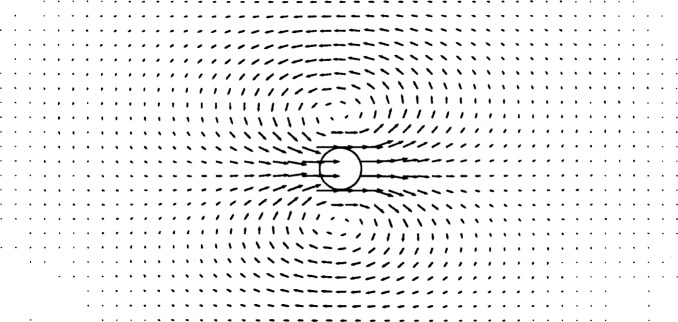
Moving particles in an otherwise stationary fluid.

To test our moving-boundary method, we ran the same simulation in the frame of reference in which the particle is moving at a constant linear velocity, −*U*_∞_, and the fluid is stationary. The resulting flow is shown in Fig. 5. This is exactly (within machine accuracy) the solution that is obtained by taking the velocity field from the previous simulation and subtracting the velocity *U*_∞_. In this frame of reference it is easier to visualize how the particle moves the fluid out of its forward path, making it circulate above and below the cylinder, pulling fluid in toward itself at the rear.

### 4.2 Stokes Flow Generated by a Rotating Cylinder

**Fig. 6:**
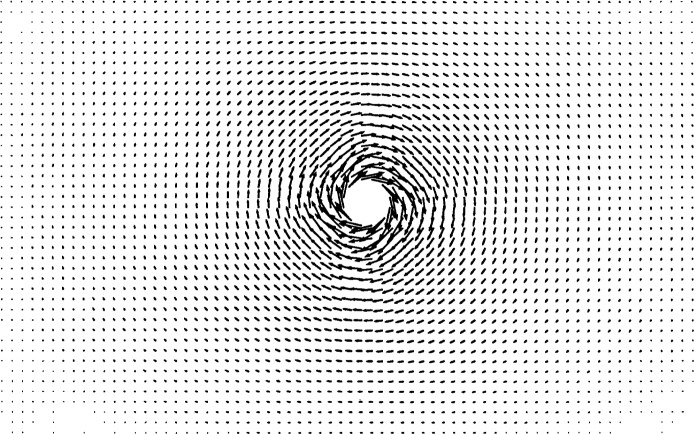
Rotating cylinder in an otherwise stationary fluid.

To test the rotational component of our model, we analyzed the axisymmetric flow generated by a right, circular cylinder rotating at a constant angular velocity. In the ideal case, the fluid velocity goes to zero asymptotically as the radial distance from the axis of symmetry goes to infinity. We used the same three finite element meshes as were used to test the model for flow around a stationary cylinder. The exact solution to this flow problem is given by Batchelor [13]:

$$U=\frac{r^{2}\Omega }{R}=\frac{r}{R},$$ (33)

in which *R* is the radius of the cylinder and *r* is the radial distance from the center of the cylinder. Figure 6 shows the resulting flow in the 50 × 25-node mesh. We calculated the RMS error of the numerical solution computed on each of the three meshes. The error decreased from 0.24 % to 0.15 % from the 1250-node mesh to the 3200-node mesh.

### 4.3 For Comparison: Stokes Flows Computed on a Fitted Mesh

**Fig. 7:**
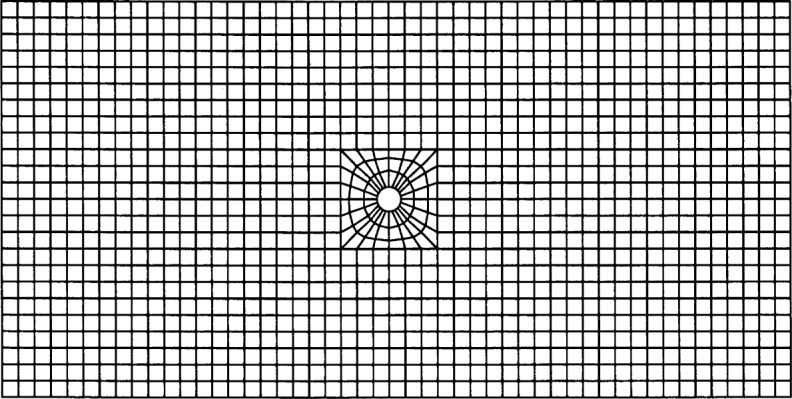
Finite element mesh representing a particle by a hole.

To compare our approach to approaches relying on remeshing, we computed solutions to both of the problems presented above on a more “traditional” finite element mesh, constructed to fit the fluid domain, including the internal boundary. This mesh is shown in Fig. 7. It has a hole in the center of it to represent the particle, and the nodes near the cylinder have been moved to accommodate the hole. This mesh is similar to the (50 × 25)-node regular mesh, but with 1297 nodes. The extra nodes were added in the process of accommodating the particle. The error in the calculation performed with the fitted mesh was 0.68 %, which is considerably greater than the 0.24 % error observed for the (50 × 25)-node mesh using our new method. This additional error is probably due to the irregular element shapes needed to form the hole in the mesh fitted. It is important to note that at least some of this discrepancy could be eliminated by optimizing the fitted mesh. However, this brings up another important point: in order to generate an accurate solution with a fitted mesh, given the necessary distortion (compared to a regular grid) to account for the hole, takes both effort and expertise. In contrast, generating the mesh for this problem using our technique is relatively trivial.

### 4.4 Stokes Flow Past a Rotating Cylinder

**Fig. 8:**
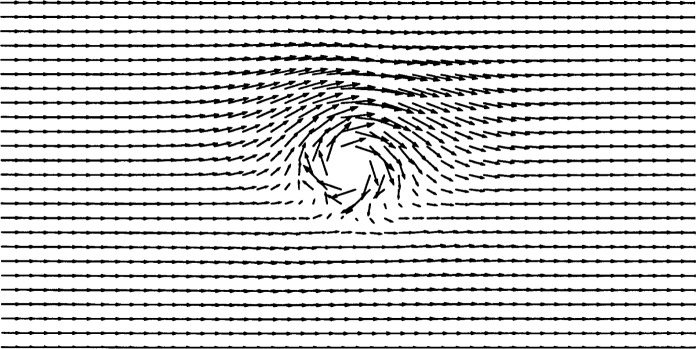
Rotating cylinder in an otherwise uniform flow field.

Figure 8 shows the flow past a rotating cylinder, the sum of the two flow fields already discussed, computed on the 1250-node mesh using our new technique. The flow accelerates as it goes over the cylinder in the direction of the rotation. This is the same flow field that is obtained by summing the velocity fields of the previous applications computed on the same mesh. This is expected, since Stokes-flow solutions are linear, and consequently, superimposable.

### 4.5 Multi-particle System

**Fig. 9:**
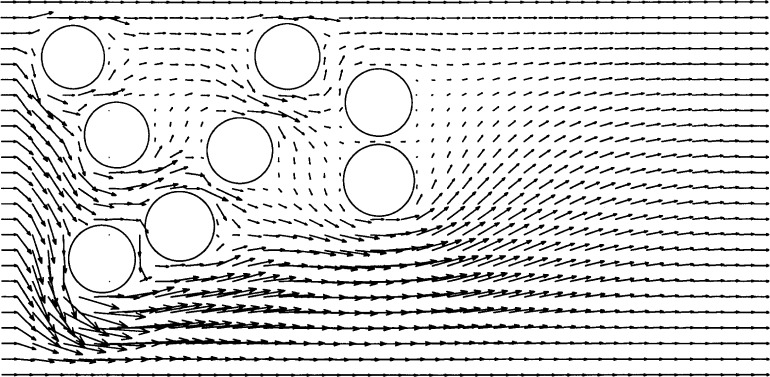
Multi-particle flow.

We computed a Navier-Stokes solution of flow through a channel around a number of particles (effectively cylinders, since we are working in two dimensions) placed rather randomly across the channel. The Reynolds number is about 1, based on a particle diameter. This illustrates the ability to place multiple objects in the flow. We selected this problem to allow a qualitative comparison with Martys and Garboczi [14]. They computed the flow field in two dimensions around roughly 100 particles using a straightforward finite difference approach on a fine grid. Our solution here appears qualitatively consistent with theirs. Figure 9 shows the flow through a channel around 8 distributed particles. The obvious result is that the dominant flow follows the path of least resistance, through the largest channels.

Multi-particle systems do not require complicated meshing schemes with our technique. We can straightforwardly model many particles without changing the background mesh, subject to the constraint of how fine the background mesh must be to capture the flow between the particles, and the corresponding limits of computer memory. The particles can be located close to one another without distorting the mesh. However, they can approach only as close as allowed by the fineness of the mesh, so that sufficient nodes remain between the particles to resolve the flow. On the coarse mesh used here, the particles are as close as allowable to maintain a qualitatively reasonable solution. Note that we do not currently allow the particles to contact each other. We have not yet accounted for particle-particle momentum transfer. This is not a major modification, and is discussed further in the Discussion and Conclusions section.

## 5. Examples—Falling Cylinders

We sought experimental data with which to validate our 2-D model. While ample literature is available on falling spheres or other particle shapes, there appears to be relatively little on multiple falling cylinders. We conducted a series of crude experiments observing up to three cylindrical metal rods falling through glycerin. The rods are initially oriented horizontal. This is a stable configuration in terms of orientation: the drag on each cylinder is such that it discourages excursions away from the horizontal. (To stray from the horizontal, one end must fall faster. However, this increases the drag on that end, providing the necessary restoring force to maintain the cylinder in a horizontal orientation.) Multiple cylinders were parallel to each other. The rods had length-to-radius ratios of 10 to minimize end effects. We conducted a few experiments with rods whose aspect ratios were 20 and saw little or no difference compared to cylinders with aspect ratios of 10. The viscosity of glycerin is large, *µ* = 11.4 g/(cm s) at 23 °C, but it behaves as a Newtonian fluid at this temperature. The density of glycerin at this temperature is 1.25 g/cm^3^. The cylinders had diameters of 0.48 cm and lengths of about 5.5 cm. The cylinders were cold-rolled steel, with a density of about 7.74 g/cm^3^. Their settling speeds approached 16 cm/s. The resulting Reynolds number is about 10 to 20 based on the diameter of the cylinders.

### 5.1 One Falling Cylinder

**Fig. 10:**
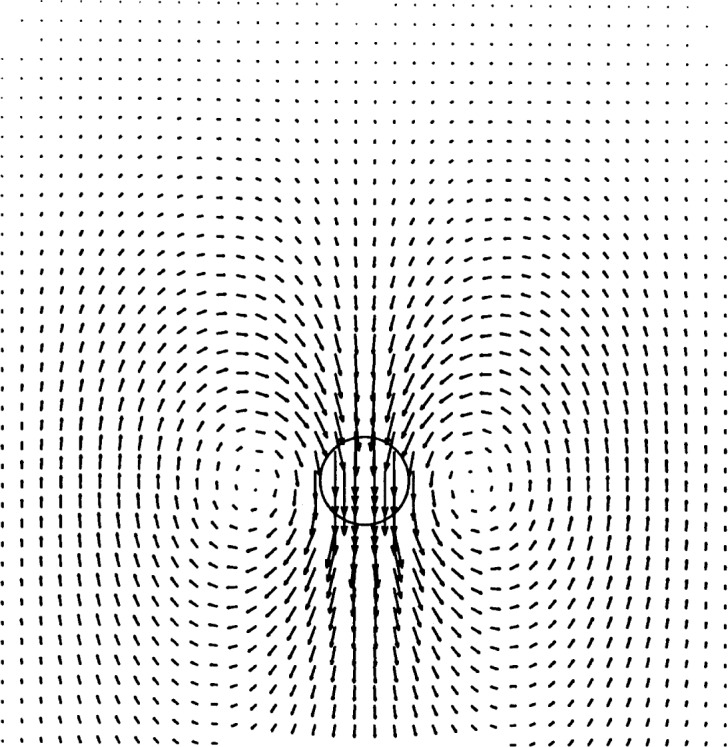
One falling cylinder.

We simulated a single particle (cylinder), initially at rest, settling under the influence of gravity. The simulation is 2-D. The boundary conditions are no-slip (zero velocity) on the top and bottom, and zero shear on the sides. This corresponds to a cylinder settling in a broad tank with a lid in contact with the liquid. In the simulation, the cylinder starts at a depth of about 1/3 of the total height of the tank, and falls about 1/3 of the height. Figure 10 shows the velocity vector-field for the flow around a single falling cylinder at a selected time step. The cylinder accelerates as it falls from its initial, stationary position. The acceleration decreases during the simulation: the drag force on the cylinder increases as its speed increases, eventually balancing the force of gravity.

An exact solution exists to determine the settling speed of a single cylinder falling in an incompressible fluid [15]. The normal and tangential stresses at the surface of the cylinder exert a drag force on the cylinder of magnitude *D*, given by the equation:

D=2πμUC, (34)

in which *C* is a constant:

$$C=\frac{2}{\ln\left(\frac{7.4}{R}\right)},$$ (35)

where *R* is represented by:

$$R=\frac{2rU\rho }{\mu }.$$ (36)

The settling speed calculated from the exact solution is *U* = 16.85 cm/s.

The simulation was run on meshes of several different grid spacings. The cylinder maintained a steady velocity only during the portion of the simulation during which it resided near the center of the mesh. Boundary effects were seen if the cylinder was too close to the upper or lower boundary. Our ability to simulate this situation was limited by the size of the mesh. The obvious cure for this problem is to translate the entire mesh at the settling speed of the particle, as was done by Johnson and Tezduyar [3]. On a domain with a grid spacing equal to 0.2 cm, on which each particle covers a 3 × 3-element area, the settling speed we computed was 15.9 cm/s, which is in error by 5.6 % compared to the exact solution. On a mesh with a 0.12 cm grid-spacing, on which each particle covers a 5 × 5-element area, the settling speed was 17.0, which is in error by 0.89 %. The method appears to converge well.

### 5.2 A Pair of Falling Cylinders

An interesting result occurs experimentally when a pair of cylinders is placed several diameters apart near the top of the glycerol and released. The two cylinders fall at the same speed. They remain parallel and side-by-side, but the distance between them increases as they fall. The cylinders are observed to rotate about their horizontal, lengthwise axes in opposing directions. Looking at the cylinders end-on, the left one rotates clockwise, while the right one rotates counterclockwise. The explanation for this is as follows. The glycerol is quite viscous, so the momentum of each cylinder diffuses quickly toward the other. The two cylinders falling side-by-side restrict the flow between them, slowing the flow there and causing the pressure to increase between the cylinders. This causes some of the glycerin lying in the path between the cylinders to flow around the outsides of the cylinders rather than between them, speeding the flow on the outsides of the cylinders. The pressure on the outsides of the cylinders is reduced, a manifestation of the Bernoulli effect. The resulting pressure gradient forces the cylinders apart. The rotation of the cylinders is the Magnus effect in reverse, where the pressure gradient (or outward diversion of the flow) causes the cylinders to counter-rotate.

**Fig. 11:**
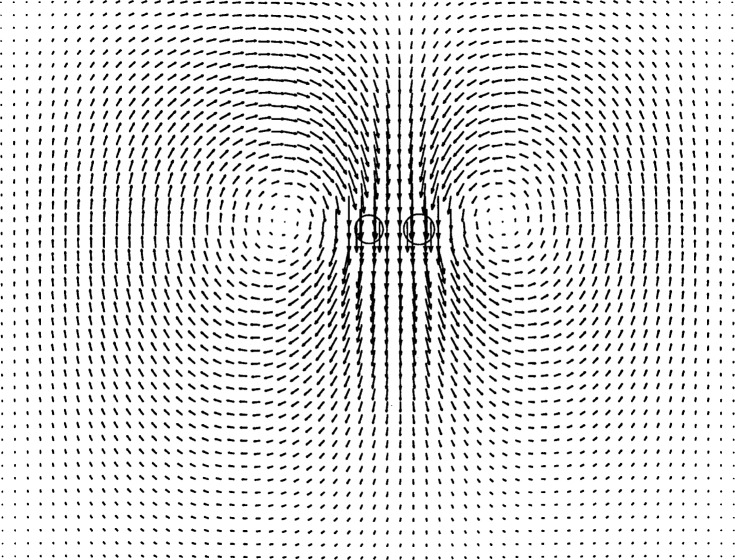
Two falling cylinders.

A finer mesh is required to reasonably simulate a pair of falling cylinders than is needed for the case of a single cylinder, because of the need to track the rotation of the particles with sufficient accuracy. Extraneous computational constraints limited the size of the mesh we were able to use. The resolution of this problem is straightforward, but is left for future work. Nonetheless, on the meshes that we used, the rotation we computed agrees qualitatively with what was observed experimentally. The cylinders fall side-by-side, moving apart and rotating in opposite directions as they descend. Figure 11 shows a snapshot of the velocity vector-field approximately midway through the simulation of two cylinders falling. The horizontal acceleration is initially zero, and increases slowly at first. The horizontal velocity components in the figure are small and not easily visible. This is another case in which it would be helpful to translate the entire mesh at the speed of the center of mass of the cylinders, as was done by Johnson and Tezduyar [3].

## 6. Discussion and Conclusions

We have created a novel technique for modeling two-phase flow of multiple particles moving in a fluid. An advantage of this approach is that the mesh does not distort as a result of the movement of the particles, obviating the need for remeshing and the associated numerical machinery. This technique is extensible to non-spherical particles. We have tested the technique by applying it to several simple linear problems for which exact solutions are known. We then illustrated the technique for cylinders falling at low Reynolds numbers. Fluid velocities computed for locations very close to particles converge toward the exact solutions as the computational mesh is refined.

An issue that has not yet been dealt with is the modeling of collisions between particles or with walls. For any technique this ultimately involves a judgment as to how close the particles may come before one considers them to contact. Physically, when the particles get too close, the assumptions of continuum mechanics are violated. However, long before that, it is a practical necessity to limit how far the calculation proceeds, because as the particles are allowed to approach more closely, the mesh must be correspondingly refined to capture the flow between the particles. The accuracy of our technique is determined by the fineness of the background mesh. The close approach of particles creates a challenge for remeshing schemes as well: frequent remeshing may be required to avoid unacceptable distortion as particles approach. We need to investigate the trade-off between the cost and accuracy of simulations as the background mesh is refined. If it turns out that too fine a mesh is required, a possibly useful compromise might be to use a hybrid scheme that uses a locally varying mesh, similar to what is used in other work, with the kind of particle definition we are using. In this way the mesh could be locally fine where necessary, but since our technique does not directly deform the mesh as the particles move it would limit the need for remeshing. We feel this technique is promising, and so have elected to present it at this time even though our numerical formulation needs to be refined to allow the finer and larger meshes required for more complex regimes of flow.

## Glossary

*g*: gravitational acceleration
*m*_p_: mass of particle
*n*: unit normal vector
*p*: pressure
*r*: radius of cylinder
*R*: radial distance from center of cylinder
*T*: temperature
***u***: velocity vector
*u_x_*: velocity in x direction
*u_y_*: velocity in y direction
*U*: magnitude of velocity
*U*_∞_: constant velocity far from particle
*x*, *y*, *z*: rectangular Cartesian coordinates
*ϵ*: penalty parameter
*Ω*: angular velocity
*µ*: viscosity
*ψ*: stream function
*ρ*: density
*σ*: shear stress
∇: gradient operator
∇·: divergence operator
∇^2^: Laplacian operator
